# Age-related heterogeneity in diagnosis-coded pediatric *Mycoplasma pneumoniae*-associated hospitalizations and PICU-associated care: a single-center retrospective study

**DOI:** 10.3389/fpubh.2026.1875440

**Published:** 2026-09-23

**Authors:** Ming Chen, Buyun Shi, Zhi Xia, Liang Bao, Xu Zhang, Simin Wang, Qin Li, Qin Hu, Wenqiang Yang

**Affiliations:** 1 Department of Dermatology, Maternal and Child Health Hospital of Hubei Province, Wuhan City, Hubei Province, China; 2 Department of Pediatric Intensive Care Unit (PICU), Maternal and Child Health Hospital of Hubei Province, Wuhan City, Hubei Province, China; 3 Children's Infectious Disease Prevention and Control Center of Hubei Province, Maternal and Child Health Hospital of Hubei Province, Wuhan City, Hubei Province, China; 4 Department of Public Health, Maternal and Child Health Hospital of Hubei Province, Wuhan City, Hubei Province, China; 5 Department of Maternal and Child Health Hospital of Hubei Province, Hubei University of Medicine, Shiyan City, Hubei Province, China

**Keywords:** children, co-diagnosis, diagnosis-coded phenotype, *Mycoplasma pneumoniae*, PICU-associated hospitalization

## Abstract

**Introduction:**

Post-pandemic re-emergence of *Mycoplasma pneumoniae* has raised concern about pediatric hospital burden and intensive-care demand. We described temporal trends, age-stratified diagnosis-coded phenotypes, pathogen-related coded co-diagnoses, and variables associated with PICU-associated hospitalization.

**Methods:**

We conducted a single-center retrospective study based on diagnosis-coded discharge records from a tertiary maternal and child health hospital in Hubei, China. Hospitalization records of children aged 29 days to <18 years with finalized diagnosis-coded *M. pneumoniae*-associated illness from June 2020 to March 2026 were screened; neonatal, adult, and manually verified repeated records were excluded. A stratified chart-validation subset (*n* = 762) summarized microbiological evidence and selected coding-derived variables.

**Results:**

The analytic dataset included 33,047 pediatric hospitalization records; 16,848 (51.0%) were male and 872 (2.6%) were PICU-associated. Monthly records peaked in November 2023 (2,282), whereas PICU-associated records and rate peaked in October 2023 (99; 4.95%). Respiratory failure/hypoxemia was recorded in 6.6% of the 1–<12 mo group versus 1.3% of the 72–<144 mo group; coded sepsis/septicemia in 11.2% versus 5.9%; and cardiac abnormality/coded myocardial involvement in 25.0% versus 15.6%. Atelectasis and consolidation peaked in the 72–<144 mo group (24.1 and 13.0%). The most common pathogen-related coded co-diagnoses were respiratory syncytial virus (2,096; 6.3%), rhinovirus (2,031; 6.1%), *Haemophilus influenzae* (1,351; 4.1%), adenovirus (1,348; 4.1%), and influenza virus (956; 2.9%). In the extended exploratory model, age 1–<12 mo (aOR, 2.59; 95% CI, 2.07–3.24), age 12–<36 mo (aOR, 1.70; 95% CI, 1.40–2.07), influenza virus (aOR, 2.07; 95% CI, 1.50–2.86), respiratory syncytial virus (aOR, 1.83; 95% CI, 1.43–2.35), and major chronic/congenital comorbidity (aOR, 8.07; 95% CI, 6.69–9.74) were associated with PICU-associated hospitalization.

**Conclusion:**

Diagnosis-coded pediatric *M. pneumoniae*-associated hospitalization records increased after June 2023 and peaked in November 2023, with age-structured coded phenotype patterns and measurable PICU-associated burden. Findings represent hospital-based diagnosis-coded patterns, not laboratory-adjudicated incident infections or population incidence.

## 1 Introduction

*Mycoplasma pneumoniae* is an important cause of respiratory tract infection and community-acquired pneumonia in children. Although many infections are self-limited, hospitalized pediatric *M. pneumoniae* pneumonia can be associated with severe or refractory disease, structural pulmonary complications, and extrapulmonary manifestations involving the skin, nervous system, cardiovascular system, gastrointestinal tract, and hematologic system (1–4). Recent evidence-based pediatric guidance in China emphasizes the use of compatible clinical features, imaging, and microbiological evidence for diagnosis, while also recognizing the role of severe and refractory phenotypes in clinical management (2).

During the COVID-19 pandemic, non-pharmaceutical interventions markedly altered circulation of respiratory pathogens. After restrictions were relaxed, *M. pneumoniae* re-emerged in several regions, with delayed but substantial post-pandemic activity reported since late 2023 (5–8). However, a single-center discharge-abstract study cannot estimate community incidence or regional prevalence. Its value lies instead in describing hospital-service burden, coding-derived phenotype structure, and severe-care demand within a consistent institutional dataset.

The interpretation of pathogen co-diagnoses is also complex. Respiratory syncytial virus (RSV) and influenza virus are major pediatric respiratory pathogens in their own right (9, 10). Respiratory pathogen co-detection in children with pneumonia may reflect overlapping infections, prolonged shedding, colonization, detection intensity, or coding practice rather than true pathogenic coinfection (11, 12). Therefore, in this study we used the term pathogen-related coded co-diagnosis for the full discharge-abstract cohort and reserved laboratory-supported co-detection for the chart-validation subset.

Using de-identified structured discharge abstracts from a tertiary maternal and child health hospital and provincial children’s infectious disease prevention and control center in Hubei, China, this study aimed to characterize monthly hospital-based burden, age-stratified diagnosis-coded phenotypes, pathogen-related coded co-diagnoses, and exploratory variables associated with PICU-associated hospitalization among diagnosis-coded pediatric *M. pneumoniae*-associated hospitalization records.

## 2 Methods

### 2.1 Study design and data source

This retrospective discharge-abstract study used a de-identified structured inpatient discharge dataset from Maternal and Child Health Hospital of Hubei Province. The dataset reflected routine hospital documentation, discharge diagnosis coding, and department-field recording. Because the primary analysis used finalized discharge abstracts rather than full clinical-chart adjudication, all phenotype and pathogen variables derived from discharge diagnosis labels were interpreted as diagnosis-coded variables. Reporting was guided by principles for observational studies using routinely collected health data, including transparency of data source, coding algorithms, and outcome definitions (13–15).

### 2.2 Study population and data cleaning

The initial screening dataset contained 34,562 diagnosis-coded *M. pneumoniae*-associated hospitalization records. Records from children aged <29 days (*n* = 55), records from patients aged ≥18 years (*n* = 1,436), and repeated hospitalization records identified through manual verification (*n* = 24) were excluded. The final analytic dataset included 33,047 eligible pediatric hospitalization records aged ≥29 days and <18 years (Supplementary Figure S1). The unit of analysis was the hospitalization record. Age was calculated at admission and expressed in month-based clinical categories: 1–<12 mo, 12–<36 mo, 36–<72 mo, 72–<144 mo, and 144–<216 mo. The 1–<12 mo group began at age 29 days because neonatal records had been excluded.

### 2.3 Microbiological and diagnosis-coded case definition

In routine institutional practice, *M. pneumoniae*-associated illness was diagnosed by treating clinicians based on compatible clinical manifestations, radiologic findings when available, and supportive laboratory evidence, including serological evidence or pathogen detection in respiratory specimens. Laboratory evidence included serology and, when available, molecular detection by polymerase chain reaction (PCR), nucleic acid amplification testing (NAAT), targeted next-generation sequencing (tNGS), or metagenomic next-generation sequencing (mNGS). This approach was consistent with pediatric guideline recommendations and current laboratory guidance supporting the combined use of clinical features and microbiological evidence, while recognizing that NAAT-based methods provide more specific diagnostic support than single-serology evidence (2, 16). In the full discharge-abstract cohort, however, records were identified from finalized diagnosis-coded discharge records rather than full laboratory adjudication. Therefore, the analytic cohort was defined as diagnosis-coded *M. pneumoniae*-associated hospitalization records, not laboratory-adjudicated incident *M. pneumoniae* infections.

### 2.4 Pathogen-related coded co-diagnoses

Pathogen-related coded co-diagnoses were derived from specific pathogen-related discharge diagnosis labels. For each pathogen group, a hospitalization record was counted once if one or more corresponding pathogen-specific diagnosis labels were present; repeated labels within the same hospitalization record were not counted more than once. Non-specific infection labels such as bacterial infection, viral infection, infectious fever, respiratory tract infection, and pulmonary infection were not included in the any-pathogen summary variable. Pertussis-like syndrome and parapertussis were not counted as pertussis. Original source-language diagnosis strings are provided for reproducibility.

### 2.5 Diagnosis-coded phenotype groups and PICU-associated hospitalization

Selected pulmonary complication, extrapulmonary, severe-care, and hematologic phenotype groups were derived from discharge diagnosis labels according to predefined grouping rules (Supplementary Table S1). Each hospitalization record was counted once for a phenotype group if one or more corresponding discharge diagnosis labels were present; repeated labels within the same hospitalization record were not counted more than once. These grouped variables were defined and interpreted as diagnosis-coded phenotype groups rather than clinically adjudicated organ involvement directly attributable to *M. pneumoniae*. For example, cardiac abnormality/coded myocardial involvement included discharge labels such as myocardial injury, myocarditis, abnormal myocardial enzyme profile, or electrocardiographic abnormality, whereas neurologic/central nervous system (CNS)-related diagnoses included neurologic or seizure-related discharge labels. The original source-language diagnosis strings and grouping rules are provided in Supplementary Table S1 for reproducibility.

PICU-associated hospitalization was defined as any hospitalization record indicating that the child received pediatric intensive care at any time during the hospital stay, based on the structured PICU field and/or department information. This definition included both direct PICU care at hospital presentation and transfer to PICU after admission to another ward. Therefore, the outcome reflects hospitalization involving PICU care rather than direct admission to the PICU at hospital presentation only.

### 2.6 Stratified chart-validation subset

To evaluate the plausibility and supporting documentation of key discharge-coded variables, we conducted a stratified systematic chart-validation substudy of 762 records. Records were stratified by admission year and PICU-associated status; within each stratum, eligible records were ordered by admission date and selected at approximately regular intervals to improve temporal coverage. The validation subset included 247 PICU-associated records and 515 non-PICU records, intentionally enriching PICU-associated records to better assess coding-derived variables related to intensive-care-associated hospitalization. The validation extraction summarized microbiological evidence for *M. pneumoniae*, chart-level support for selected coded phenotype and comorbidity variables, documentation of other respiratory pathogen testing, and PICU-associated care reasons among PICU-associated validation records. Because PICU-associated records were intentionally enriched, chart-validation findings were interpreted as validation summaries rather than unweighted full-cohort prevalence estimates.

### 2.7 Statistical analysis

Categorical variables were summarized as counts and percentages, and continuous variables were summarized as medians with interquartile ranges (IQRs). Age-group comparisons were performed using chi-square tests for categorical variables and Kruskal–Wallis tests for continuous variables. Because of the large sample size and multiple descriptive comparisons, *p* values were interpreted as exploratory, and interpretation emphasized absolute age-stratified differences rather than statistical significance alone. Monthly hospitalization counts, monthly PICU-associated records, and monthly PICU-associated rates were calculated descriptively.

Logistic regression models were used to estimate adjusted odds ratios (aORs) and 95% confidence intervals (CIs) for PICU-associated hospitalization. The parsimonious model included age group, sex, and selected common pathogen-related coded co-diagnoses, including rhinovirus, *Haemophilus influenzae*, adenovirus, respiratory syncytial virus, and influenza virus. The extended exploratory model additionally adjusted for major chronic/congenital comorbidity, admission year, and season as categorical covariates. Season was assigned according to admission month: spring (March–May), summer (June–August), autumn (September–November), and winter (December–February). The reference categories in the extended model were 72–<144 mo for age group, 2024 for admission year, and autumn for season. Full model estimates and model summaries were reported with the model results. The models were intended to explore associations based on available coded variables rather than identify causal risk factors or construct a definitive clinical prediction model.

All statistical tests were two-sided. Analyses were performed in Python version 3.11.2 using pandas, NumPy, SciPy, and statsmodels; figures were generated using Matplotlib.

## 3 Results

### 3.1 Study population

The final analytic dataset included 33,047 pediatric diagnosis-coded *M. pneumoniae*-associated hospitalization records. There were 2,882 records in the 1–<12 mo group, 7,824 in the 12–<36 mo group, 10,747 in the 36–<72 mo group, 10,966 in the 72–<144 mo group, and 628 in the 144–<216 mo group. Overall, 16,848 records (51.0%) were from male patients, 872 (2.6%) were PICU-associated, and 4,100 (12.4%) had a hospital stay longer than 7 days. The median age at admission was 52.7 months (IQR, 26.5–84.8), and the median length of stay was 5 days (IQR, 4–6). Specific pathogen-related coded co-diagnoses were recorded in 9,516 records (28.8%) (Table 1).

**Table 1:** Cohort characteristics by age group.

Characteristic	Overall	1–<12 mo^*^	12–<36 mo	36–<72 mo	72–<144 mo	144–<216 mo	*p* value
Hospitalization records, *n*	33,047	2,882	7,824	10,747	10,966	628	
Male sex	16,848 (51.0%)	1739 (60.3%)	4,111 (52.5%)	5,195 (48.3%)	5,501 (50.2%)	302 (48.1%)	<0.001
Female sex	16,199 (49.0%)	1,143 (39.7%)	3,713 (47.5%)	5,552 (51.7%)	5,465 (49.8%)	326 (51.9%)	<0.001
PICU-associated hospitalization	872 (2.6%)	153 (5.3%)	229 (2.9%)	239 (2.2%)	236 (2.2%)	15 (2.4%)	<0.001
Hospital stay >7 days	4,100 (12.4%)	444 (15.4%)	891 (11.4%)	1,287 (12.0%)	1,389 (12.7%)	89 (14.2%)	<0.001
Any pathogen-related coded co-diagnosis	9,516 (28.8%)	1,088 (37.8%)	2,665 (34.1%)	3,079 (28.6%)	2,551 (23.3%)	133 (21.2%)	<0.001
Age at admission, months, median (IQR)	52.7 (26.5–84.8)	8.3 (6.2–10.2)	21.8 (16.6–28.5)	51.4 (43.4–60.9)	93.6 (82.7–109.5)	155.6 (148.8–165.4)	<0.001
Length of stay, days, median (IQR)	5 (4–6)	5 (4–7)	5 (4–6)	5 (4–6)	5 (4–6)	5 (4–6)	<0.001

Values are counts (%) unless otherwise indicated. Percentages were calculated within each age group. *p* values were obtained using chi-square tests for categorical variables and Kruskal–Wallis tests for continuous variables and should be interpreted as exploratory. The unit of analysis was the hospitalization record. Age groups were assigned using exact age in days and expressed as month-equivalent categories; the 1–<12 mo group began at age 29 days because neonatal records were excluded. Any pathogen-related coded co-diagnosis was defined using specific pathogen-related discharge diagnosis labels and excluded non-specific infection labels. PICU, pediatric intensive care unit; IQR, interquartile range; mo, months. *The 1–<12 mo group began at age 29 days because neonatal records were excluded.

### 3.2 Monthly hospitalization records and PICU-associated burden

Monthly diagnosis-coded hospitalization records remained relatively low during 2020–2022, ranging from 20 to 536 records per month. In 2023, monthly records increased from 99 in January and 139 in February to 418 in March, 531 in May, 751 in June, and 1,102 in July, then reached 2,001 in October and peaked at 2,282 in November. Monthly PICU-associated records and the PICU-associated rate peaked 1 month earlier, in October 2023, at 99 records and 4.95%, respectively (Figure 1; Supplementary Table S2). These trends describe hospital-based discharge records and should not be interpreted as population incidence.

**Figure 1:**
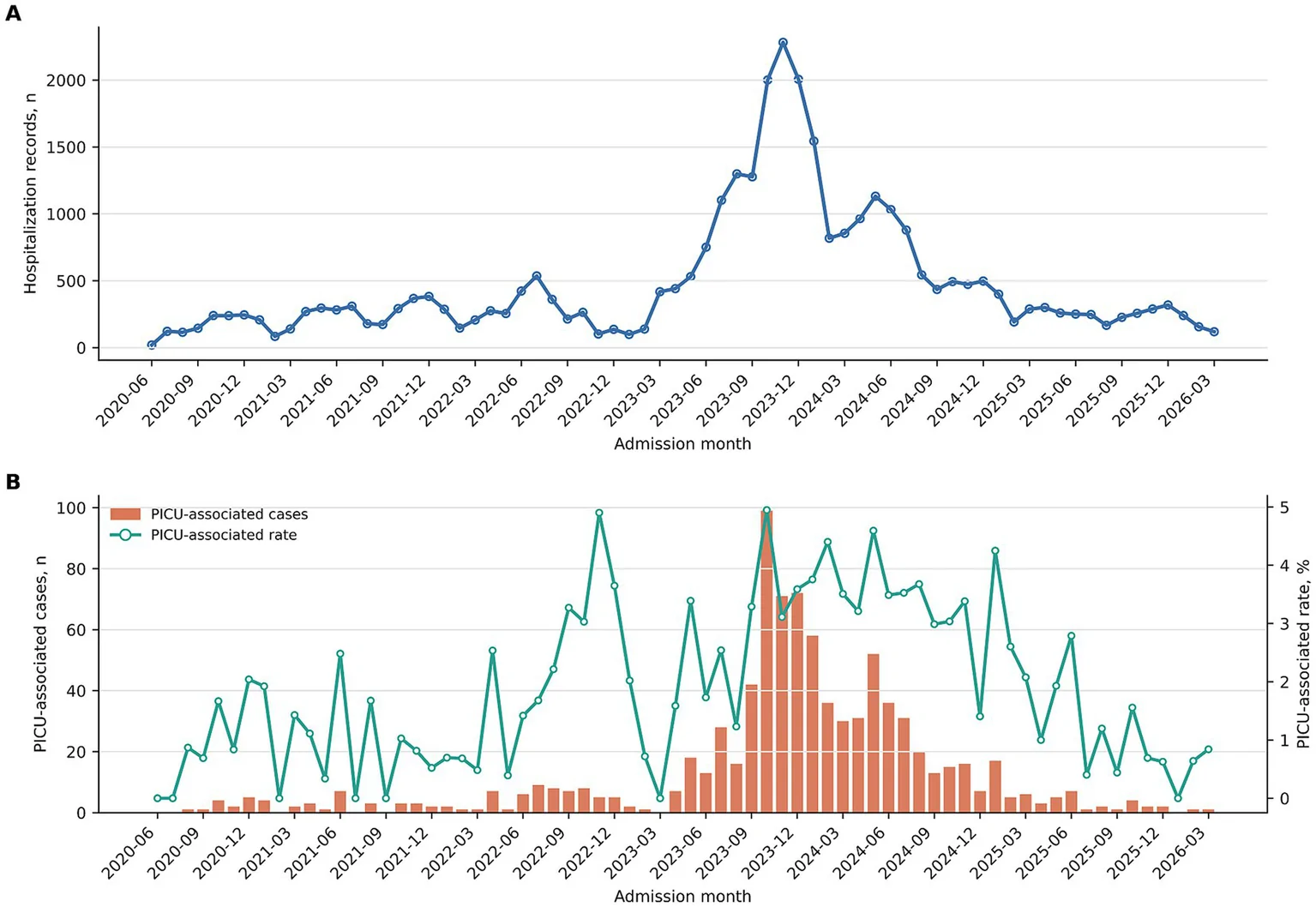
Monthly diagnosis-coded *M. pneumoniae-ass*ociated hospitalization records and PICU-associated burden, June 2020 to March 2026. **(A)** Monthly total diagnosis-coded *M. pneumoniae-ass*ociated hospitalization records. **(B)** Monthly PICU-associated records and monthly PICU-associated rate. The PICU-associated rate was calculated as the number of PICU-associated hospitalization records divided by the total number of *M. pneumoniae-ass*ociated hospitalization records in the corresponding month. These trends represent hospital-based discharge records and should not be interpreted as population incidence. PICU, Pediatric intensive care unit.

### 3.3 Age-stratified diagnosis-coded phenotype groups

Age-stratified analysis showed substantial heterogeneity in selected diagnosis-coded phenotype groups (Figure 2; Table 2). Respiratory failure/hypoxemia was recorded in 6.6% of the 1–<12 mo group and 3.3% of the 12–<36 mo group, compared with 1.3% of the 72–<144 mo group. Coded sepsis/septicemia was recorded in 11.2 and 10.4% of the 1–<12 mo and 12–<36 mo groups, respectively, compared with 5.9% in the 72–<144 mo group. Cardiac abnormality/coded myocardial involvement was also more frequent in younger children, occurring in 25.0% of the 1–<12 mo group and 23.0% of the 12–<36 mo group, compared with 15.6% of the 72–<144 mo group. Neurologic/CNS-related diagnoses were most frequent in the 12–<36 mo group (7.1%). In contrast, structural pulmonary complication labels were more prominent in the 72–<144 mo group, with atelectasis and consolidation peaking in this group at 24.1 and 13.0%, respectively.

**Figure 2:**
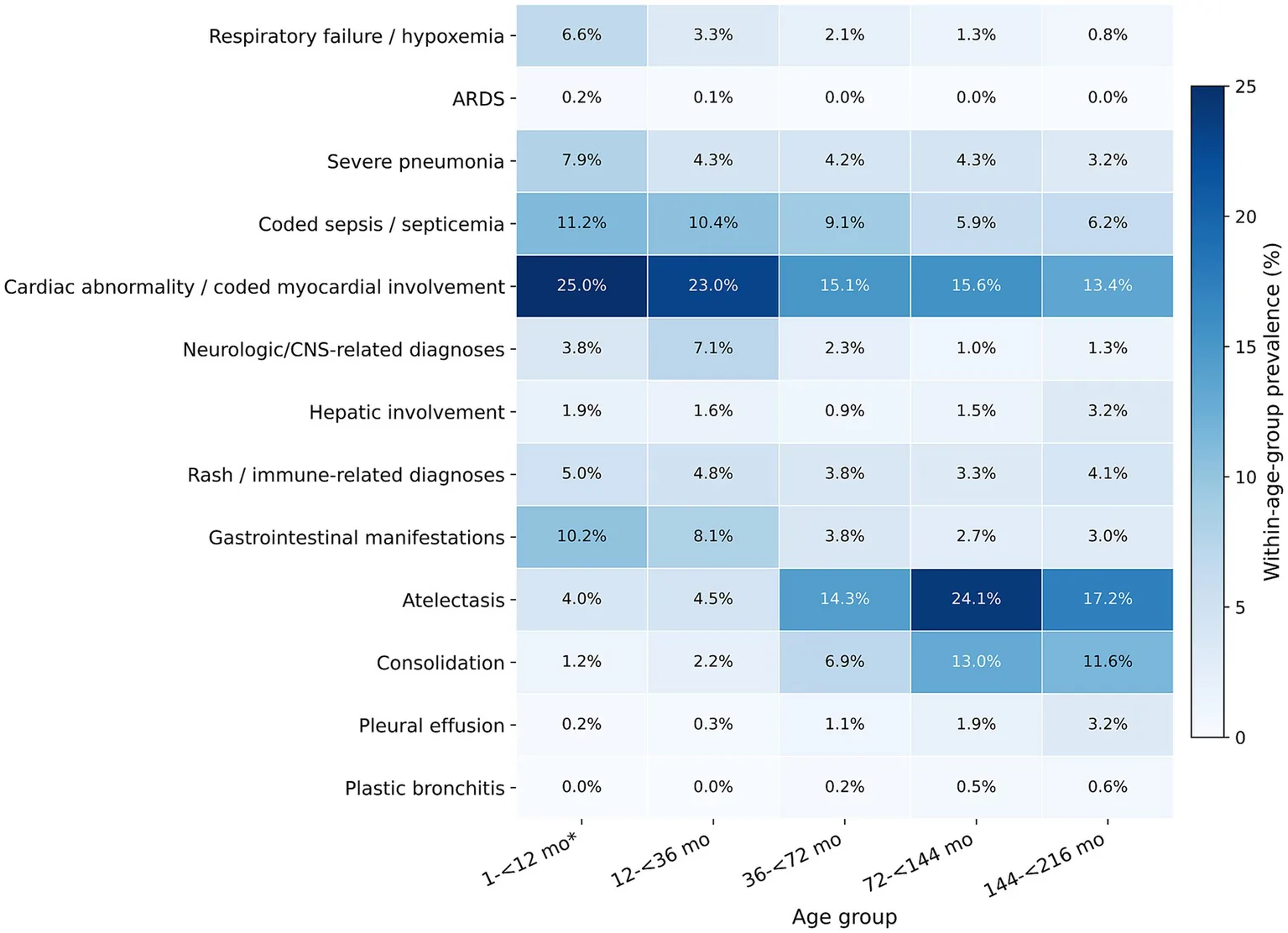
Age-stratified prevalence of selected diagnosis-coded phenotype groups. The heatmap shows the within-age-group percentage of hospitalization records with each selected diagnosis-coded phenotype group. Phenotype groups were derived from discharge diagnosis labels and should be interpreted as diagnosis-coded phenotype groups rather than clinically adjudicated organ involvement directly attributable to *M. pneumoniae*. The 1–<12 mo group began at age 29 days because neonatal records were excluded. ARDS, acute respiratory distress syndrome; CNS, central nervous system; mo, months.

**Table 2:** Age-stratified selected diagnosis-coded phenotype groups.

Diagnosis-coded phenotype group	Overall	1–<12 mo*	12–<36 mo	36–<72 mo	72–<144 mo	144–<216 mo	*p* value
Respiratory failure/hypoxemia	829 (2.5%)	191 (6.6%)	258 (3.3%)	230 (2.1%)	145 (1.3%)	5 (0.8%)	<0.001
ARDS	13 (0.0%)	5 (0.2%)	5 (0.1%)	2 (0.0%)	1 (0.0%)	0 (0.0%)	0.001
Severe pneumonia	1,501 (4.5%)	227 (7.9%)	335 (4.3%)	451 (4.2%)	468 (4.3%)	20 (3.2%)	<0.001
Coded sepsis/septicemia	2,805 (8.5%)	324 (11.2%)	815 (10.4%)	982 (9.1%)	645 (5.9%)	39 (6.2%)	<0.001
Cardiac abnormality/coded myocardial involvement	5,938 (18.0%)	720 (25.0%)	1797 (23.0%)	1,628 (15.1%)	1709 (15.6%)	84 (13.4%)	<0.001
Neurologic/CNS-related diagnoses	1,027 (3.1%)	110 (3.8%)	553 (7.1%)	242 (2.3%)	114 (1.0%)	8 (1.3%)	<0.001
Hepatic involvement	454 (1.4%)	54 (1.9%)	122 (1.6%)	96 (0.9%)	162 (1.5%)	20 (3.2%)	<0.001
Rash/immune-related diagnoses	1,317 (4.0%)	143 (5.0%)	375 (4.8%)	412 (3.8%)	361 (3.3%)	26 (4.1%)	<0.001
Gastrointestinal manifestations	1,651 (5.0%)	294 (10.2%)	637 (8.1%)	404 (3.8%)	297 (2.7%)	19 (3.0%)	<0.001
Atelectasis	4,756 (14.4%)	114 (4.0%)	355 (4.5%)	1,534 (14.3%)	2,645 (24.1%)	108 (17.2%)	<0.001
Consolidation	2,449 (7.4%)	35 (1.2%)	173 (2.2%)	739 (6.9%)	1,429 (13.0%)	73 (11.6%)	<0.001
Pleural effusion	370 (1.1%)	7 (0.2%)	24 (0.3%)	116 (1.1%)	203 (1.9%)	20 (3.2%)	<0.001
Plastic bronchitis	85 (0.3%)	0 (0.0%)	2 (0.0%)	25 (0.2%)	54 (0.5%)	4 (0.6%)	<0.001

Values are counts (%) within each age group unless otherwise indicated. Phenotype groups were derived from discharge diagnosis labels and should be interpreted as diagnosis-coded phenotype groups rather than clinically adjudicated organ involvement directly attributable to *M. pneumoniae*. Broad categories such as coded sepsis/septicemia and cardiac abnormality/coded myocardial involvement represent discharge-label frequencies rather than standardized clinical adjudication. *p* values were obtained using chi-square tests and should be interpreted as exploratory. ARDS, acute respiratory distress syndrome; CNS, central nervous system; mo, months. *The 1–<12 mo group began at age 29 days because neonatal records were excluded.

### 3.4 Pathogen-related coded co-diagnoses

In the full cohort, the most common specific pathogen-related coded co-diagnosis groups were respiratory syncytial virus (2,096; 6.3%), rhinovirus (2,031; 6.1%), *Haemophilus influenzae* (1,351; 4.1%), adenovirus (1,348; 4.1%), influenza virus (956; 2.9%), parainfluenza virus (803; 2.4%), Streptococcus/Pneumococcus (708; 2.1%), Epstein–Barr virus (614; 1.9%), Chlamydia spp. (444; 1.3%), and pertussis (268; 0.8%) (Table 3). The complete pathogen-related coded diagnosis spectrum, including less frequent pathogen-specific labels, is provided in Supplementary Table S3. The most frequent exact non-Mycoplasma co-diagnosis terms are provided in Supplementary Table S4.

**Table 3:** Selected pathogen-related coded co-diagnosis labels according to PICU-associated hospitalization status.

Pathogen-related coded co-diagnosis label	Overall	PICU-associated hospitalization	Non-PICU hospitalization	*p* value
Respiratory syncytial virus	2096 (6.3%)	87 (10.0%)	2009 (6.2%)	<0.001
Rhinovirus	2031 (6.1%)	48 (5.5%)	1983 (6.2%)	0.467
*Haemophilus influenzae*	1,351 (4.1%)	37 (4.2%)	1,314 (4.1%)	0.883
Adenovirus	1,348 (4.1%)	43 (4.9%)	1,305 (4.1%)	0.229
Influenza virus	956 (2.9%)	45 (5.2%)	911 (2.8%)	<0.001
Parainfluenza virus	803 (2.4%)	31 (3.6%)	772 (2.4%)	0.038
Streptococcus/Pneumococcus	708 (2.1%)	28 (3.2%)	680 (2.1%)	0.037
Epstein–Barr virus	614 (1.9%)	14 (1.6%)	600 (1.9%)	0.665
Chlamydia spp.	444 (1.3%)	17 (1.9%)	427 (1.3%)	0.154
Pertussis	268 (0.8%)	13 (1.5%)	255 (0.8%)	0.038

Values are counts (%) unless otherwise indicated. Percentages in subgroup columns were calculated within the corresponding PICU-associated or non-PICU hospitalization subgroup. Each pathogen-related coded co-diagnosis group was counted once per hospitalization record if one or more corresponding specific pathogen-related discharge diagnosis labels were present. These variables represent pathogen-related coded co-diagnoses rather than systematic laboratory co-detection or confirmed causative coinfection. *p* values were obtained using chi-square tests and should be interpreted as exploratory. PICU, pediatric intensive care unit.

### 3.5 Exploratory model for PICU-associated hospitalization

The parsimonious exploratory model including age group, sex, and selected pathogen-related coded co-diagnoses is provided in Supplementary Table S5. Major chronic/congenital comorbidity categories are summarized in Supplementary Table S6. In the mutually adjusted extended exploratory model, age 1–<12 mo (aOR, 2.59; 95% CI, 2.07–3.24), age 12–<36 mo (aOR, 1.70; 95% CI, 1.40–2.07), respiratory syncytial virus (aOR, 1.83; 95% CI, 1.43–2.35), influenza virus (aOR, 2.07; 95% CI, 1.50–2.86), and any major chronic/congenital comorbidity (aOR, 8.07; 95% CI, 6.69–9.74) were associated with PICU-associated hospitalization. The extended model included age group, sex, selected pathogen-related coded co-diagnoses, major chronic/congenital comorbidity, admission year, and season (Figure 3; Supplementary Table S7A). Model summaries are provided in Supplementary Table S7B. These associations should be interpreted as discharge-coded risk-stratification signals rather than independent causal predictors.

**Figure 3:**
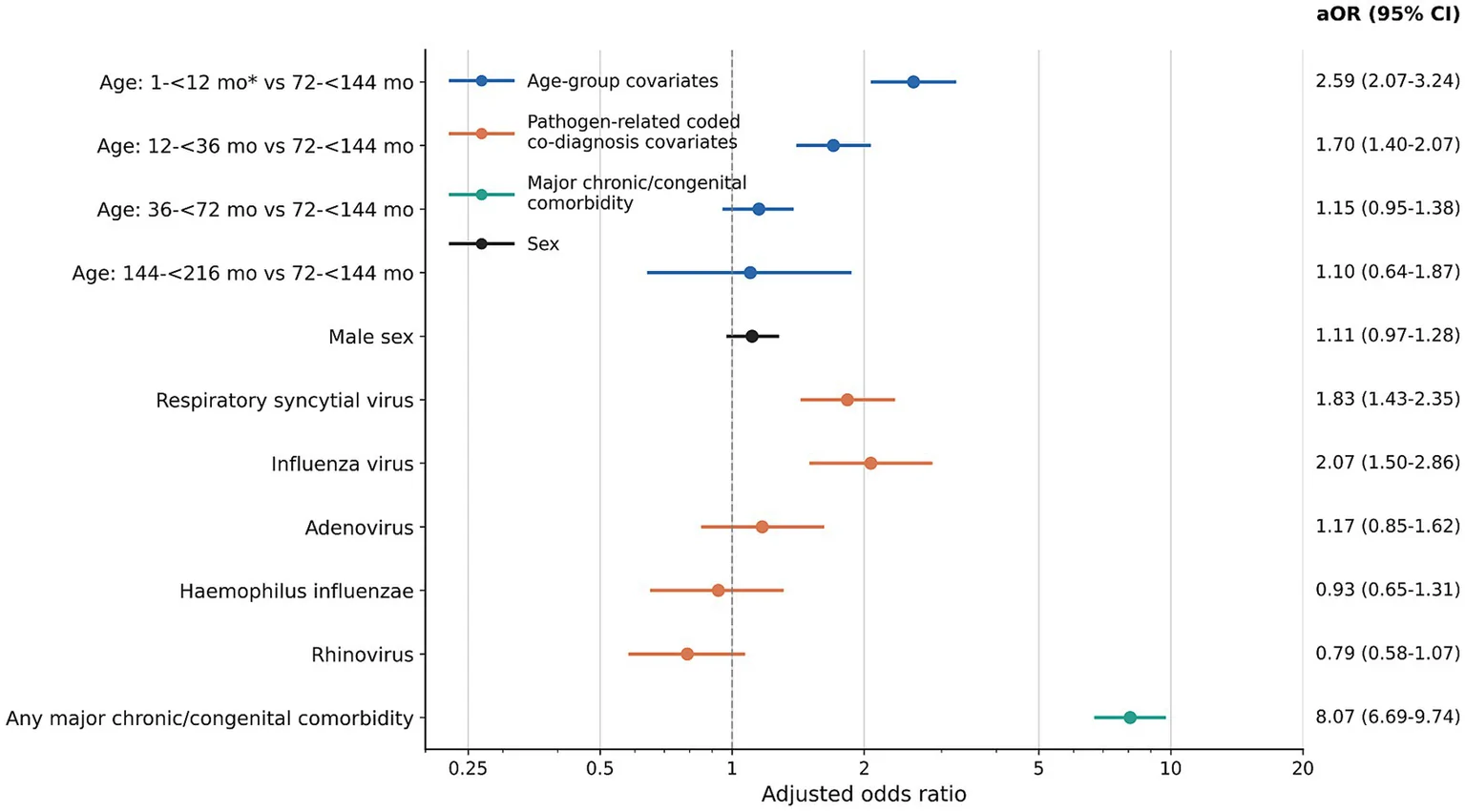
Extended exploratory logistic regression model for PICU-associated hospitalization. Points indicate adjusted odds ratios, and horizontal bars indicate 95% confidence intervals. The vertical dashed line indicates an adjusted odds ratio of 1. The displayed estimates are selected variables from the extended exploratory model; the full model additionally adjusted for admission year and season and is reported in Supplementary Table S7A. The reference age group was 72–<144 mo. The model was intended to explore associations based on available coded variables and should not be interpreted as a causal prediction model. aOR, adjusted odds ratio; CI, confidence interval; PICU, pediatric intensive care unit; mo, months.

### 3.6 Stratified chart-validation subset

In the 762-record chart-validation subset, microbiological evidence for *M. pneumoniae* was documented for all sampled records. Molecular evidence was present in 737 records (96.7%), including PCR/NAAT evidence in 565 (74.1%), tNGS evidence in 136 (17.8%), and mNGS evidence in 36 (4.7%). Supportive single-serology evidence was documented in 715 records (93.8%), and paired serology or four-fold rise was recorded in 25 (3.3%) (Supplementary Table S8). Chart review supported 761/762 (99.9%) selected coded pulmonary/extrapulmonary phenotype variables and showed concordance for 722/762 (94.8%) major chronic/congenital comorbidity coding entries. PICU-associated care reasons were summarized only among the PICU-associated validation subset (Supplementary Tables S9A, S9B).

## 4 Discussion

This single-center discharge-abstract study identified three major findings. First, diagnosis-coded pediatric *M. pneumoniae*-associated hospitalization records increased markedly from June 2023 onward, with the highest monthly total observed in November 2023, and the highest monthly PICU-associated burden preceded the peak in total monthly records. Second, age structured the distribution of coded phenotypes: younger children had higher proportions of severe-care and systemic labels, whereas school-aged children had higher proportions of structural pulmonary complication labels. Third, selected pathogen-related coded co-diagnoses and major chronic/congenital comorbidity were associated with PICU-associated hospitalization in an exploratory model.

This study adds a hospital-service perspective based on routinely collected discharge abstracts to the literature on pediatric *M. pneumoniae* re-emergence. Clinically adjudicated cohorts are essential for defining laboratory-confirmed infection, imaging severity, treatment, and individual outcomes. In contrast, our study used a large single-center discharge-abstract dataset to describe monthly diagnosis-coded hospitalization records, age-stratified coding-derived phenotype patterns, pathogen-related coded co-diagnoses, and PICU-associated care. Thus, the principal contribution is not to estimate population incidence, prove acute infection in every record, or attribute organ involvement causally to *M. pneumoniae*, but to show how diagnosis-coded hospitalizations and severe-care demand were distributed over time and across age groups within a consistent tertiary pediatric service system during the post-pandemic period.

The temporal pattern is consistent with international reports of delayed post-pandemic re-emergence of *M. pneumoniae*, including recent hospital-based pediatric data and surveillance reports (5–8, 17). A recent Italian pediatric tertiary-center study of laboratory-confirmed *M. pneumoniae* infection from 2017 to 2024 reported a marked post-pandemic increase in MP-related hospitalizations, whereas respiratory support requirements, PICU-related outcomes, and length of stay remained broadly comparable with the pre-pandemic period (7). That clinically adjudicated cohort provides an important clinical reference for interpreting post-pandemic hospital burden, but it differs from the present study in data structure and inference: the Italian study evaluated microbiologically confirmed infection episodes with clinical, laboratory, and radiologic information, whereas our study describes diagnosis-coded hospitalization records and hospital-service burden in a large discharge-abstract dataset. Therefore, our findings complement rather than replicate clinically adjudicated MP cohorts. They should be interpreted as hospital-based diagnosis-coded patterns rather than population incidence or laboratory-confirmed infection incidence. The Centers for Disease Control and Prevention (CDC) also notes that *M. pneumoniae* infection is not nationally notifiable and that surveillance relies on syndromic, laboratory, outbreak, and special-study data sources (17), further supporting a cautious interpretation of our hospital-based temporal pattern.

The age-stratified phenotype pattern should be interpreted as a diagnosis-coded pattern rather than a directly adjudicated biological phenotype. The grouped variables were derived from discharge diagnosis labels and may reflect clinical disease, testing intensity, age-related documentation, clinical coding terms, and referral pathways. This concern is particularly relevant for cardiac abnormality/coded myocardial involvement, neurologic/CNS-related diagnoses, and coded sepsis/septicemia, because these categories included broad discharge-diagnosis labels rather than standardized adjudication of organ involvement. Nevertheless, the observed age-structured distribution remains clinically informative when interpreted within these boundaries. Younger children may have lower respiratory reserve and different thresholds for systemic diagnostic labeling or escalation of care, whereas school-aged children with *M. pneumoniae*-associated pneumonia may more often show structural pulmonary complication labels such as atelectasis and consolidation. Prior pediatric studies have described severe or refractory *M. pneumoniae* pneumonia, immune activation, mucus plug formation, macrolide-resistant infection, and cardiac or other extrapulmonary manifestations, supporting the biological plausibility of age-related heterogeneity while also emphasizing the need for standardized clinical, imaging, biomarker, and microbiological definitions in future studies (18–22). In line with reporting principles for studies using routinely collected health data, we therefore emphasized transparent coding rules, source-language diagnosis strings, and cautious interpretation of coding-derived variables (13–15).

The pathogen-related co-diagnosis findings require careful distinction between coded co-diagnosis, laboratory co-detection, and true causative coinfection. In the full cohort, we analyzed discharge diagnosis labels rather than systematic laboratory co-detection across all children. In the chart-validation subset, microbiological evidence was summarized at the record level; however, even molecular detection may reflect asymptomatic carriage, prolonged detection, timing of sampling, or detection of a pathogen that was not the primary driver of severity (7, 23). This distinction is particularly important for RSV and influenza virus, because both pathogens can independently cause severe pediatric respiratory disease (9, 10). Recent pediatric studies have shown that respiratory virus or adenovirus co-detection in *M. pneumoniae* pneumonia may be clinically relevant, but co-detection alone does not establish etiologic causality, infection timing, pathogen burden, or directionality of severity (12, 24–27). Accordingly, the associations between pathogen-related coded co-diagnoses and PICU-associated hospitalization in our dataset should be interpreted as discharge-coded risk signals rather than evidence of causal coinfection.

The multivariable findings are best interpreted as exploratory associations based on available coded variables rather than independent causal predictors or a definitive clinical prognostic model. Although the extended model adjusted for age, sex, selected pathogen-related coded co-diagnoses, major chronic/congenital comorbidity, admission year, and season, the full cohort did not contain standardized radiologic severity scores, inflammatory biomarkers, oxygen requirement before PICU transfer, treatment intensity, macrolide-resistance results, or pathogen load. These factors have been evaluated in clinically adjudicated pediatric *M. pneumoniae* studies and may influence severity, structural pulmonary complications, extrapulmonary involvement, or treatment response (18–22). Therefore, the model should not be used to infer that RSV, influenza virus, or comorbidity causally determined PICU-associated hospitalization. Rather, it should be viewed as a discharge-coded risk-stratification analysis showing which routinely available demographic, comorbidity, calendar-time, and pathogen-coded variables were associated with PICU-associated care in this hospital-based dataset.

Several limitations should be considered. First, this was a single-center study conducted in a tertiary maternal and child health hospital and provincial children’s infectious disease center, and the findings may not be generalizable to community hospitals or population-level surveillance settings. Second, the primary dataset was based on structured discharge abstracts; therefore, diagnosis-coded phenotype groups may be influenced by clinical documentation, testing intensity, coding conventions, and referral pathways. Because the study was designed to cover nearly 6 years and more than 33,000 hospitalization records, full manual extraction of item-level laboratory results, imaging findings, treatment processes, pathogen loads, and macrolide-resistance testing for every record was not feasible. To partially address this limitation, we conducted a stratified chart-validation substudy of 762 records, intentionally including an enriched PICU-associated subset, to summarize microbiological evidence and assess selected coding-derived variables. However, this validation subset was not intended to replace full-cohort clinical adjudication or to estimate unweighted full-cohort prevalence of chart-level findings. Third, *M. pneumoniae* diagnostic practices and available microbiological methods may have changed over time, and full-cohort modality-level diagnostic data were unavailable. Fourth, single-serology evidence was common in the validation subset and should be treated as supportive rather than definitive evidence. Fifth, pathogen-related coded co-diagnoses cannot establish infection timing, pathogen burden, or causative coinfection. Finally, the study did not evaluate mortality as a primary outcome and did not include complete treatment, resistance, or longitudinal follow-up data. These limitations are common in studies based on routinely collected health data and reinforce the need for transparent coding algorithms, chart-validation summaries, and cautious interpretation of discharge-coded findings (13–15).

## 5 Conclusion

In this large single-center discharge-abstract cohort, diagnosis-coded pediatric *M. pneumoniae*-associated hospitalization records rose above the 2020–2022 monthly range from June 2023 and peaked in November 2023, with age-structured diagnosis-coded phenotype patterns and measurable PICU-associated burden. The findings may inform hospital-based monitoring systems that consider age structure, coding-derived phenotype patterns, pathogen-related coded co-diagnoses, and intensive-care resource use. They should be interpreted as diagnosis-coded hospital-service patterns rather than laboratory-adjudicated incident infections or population incidence.

## Data Availability

The data used in this study were derived from hospital discharge records and are not publicly available because they are subject to institutional access restrictions and may contain potentially identifiable health information. De-identified analytic code, grouping rules, and aggregated tables may be made available by the corresponding author upon reasonable request and with approval from the relevant institution.

## References

[1] Waites KB Xiao L Liu Y Balish MF Atkinson TP. *Mycoplasma pneumoniae* from the respiratory tract and beyond. Clin Microbiol Rev. (2017) 30:747–809. doi: 10.1128/CMR.00114-16, 28539503 PMC5475226

[2] Subspecialty Group of Respiratory, Society of Pediatrics, Chinese Medical Association, China National Clinical Research Center of Respiratory Diseases. Evidence-based guideline for the diagnosis and treatment of *Mycoplasma pneumoniae* pneumonia in children (2023). Pediatr Investig. (2025) 9:1–11. doi: 10.1002/ped4.12469, 40241891 PMC11998179

[3] Biagi C Cavallo A Rocca A Dondi A Salvini F Andreozzi L . Pulmonary and extrapulmonary manifestations in hospitalized children with *Mycoplasma pneumoniae* infection. Microorganisms. (2021) 9:2553. doi: 10.3390/microorganisms9122553, 34946154 PMC8707942

[4] Meyer Sauteur PM Ramien ML Theiler M. *Mycoplasma pneumoniae*: extrapulmonary manifestations in children with focus on mucocutaneous disease. Pediatr Infect Dis J. (2025) 44:e454–8. doi: 10.1097/INF.0000000000004946, 40839886

[5] Meyer Sauteur PM Beeton ML. *Mycoplasma pneumoniae*: delayed re-emergence after COVID-19 pandemic restrictions. Lancet Microbe. (2024) 5:e100–1. doi: 10.1016/S2666-5247(23)00344-0, 38008103

[6] Li H Li S Yang H Chen Z Zhou Z. Resurgence of *Mycoplasma pneumoniae* by macrolide-resistant epidemic clones in China. Lancet Microbe. (2024) 5:e515. doi: 10.1016/S2666-5247(23)00405-6, 38244553

[7] Bianchi M Pisani M Ricotta L D'Amore C Vittucci AC Cristaldi S . Epidemiology and clinical impact of *Mycoplasma pneumoniae* in an Italian pediatric center: an observational study from 2017 to 2024. Pediatr Infect Dis J. (2026) 45:132–9. doi: 10.1097/INF.0000000000004993, 40966764

[8] Diaz MH Hersh AL Olson J Shah SS Hall M Edens C. *Mycoplasma pneumoniae* infections in hospitalized children—United States, 2018-2024. MMWR Morb Mortal Wkly Rep. (2025) 74:394–400. doi: 10.15585/mmwr.mm7423a1, 40569891 PMC12200605

[9] Wang X Li Y O'Brien KL Madhi SA Widdowson MA Byass P . Global burden of respiratory infections associated with seasonal influenza in children under 5 years in 2018. Lancet Glob Health. (2020) 8:e497–510. doi: 10.1016/S2214-109X(19)30545-5, 32087815 PMC7083228

[10] Li Y Wang X Blau DM Caballero MT Feikin DR Gill CJ . Global, regional, and national disease burden estimates of acute lower respiratory infections due to respiratory syncytial virus in children younger than 5 years in 2019: a systematic analysis. Lancet. (2022) 399:2047–64. doi: 10.1016/S0140-6736(22)00478-0, 35598608 PMC7613574

[11] Jain S Williams DJ Arnold SR Ampofo K Bramley AM Reed C . Community-acquired pneumonia requiring hospitalization among U.S. children. N Engl J Med. (2015) 372:835–45. doi: 10.1056/NEJMoa1405870, 25714161 PMC4697461

[12] Dong X Li R Zou Y Chen L Zhang H Lyu F . Co-detection of respiratory pathogens in children with *Mycoplasma pneumoniae* pneumonia: a multicenter study. Front Pediatr. (2025) 13:1482880. doi: 10.3389/fped.2025.1482880, 40487016 PMC12141229

[13] von Elm E Altman DG Egger M Pocock SJ Gøtzsche PC Vandenbroucke JP. The strengthening the reporting of observational studies in epidemiology (STROBE) statement: guidelines for reporting observational studies. PLoS Med. (2007) 4:e296. doi: 10.1371/journal.pmed.0040296, 17941714 PMC2020495

[14] Benchimol EI Smeeth L Guttmann A Harron K Moher D Petersen I . The REporting of studies conducted using observational routinely-collected health data (RECORD) statement. PLoS Med. (2015) 12:e1001885. doi: 10.1371/journal.pmed.1001885, 26440803 PMC4595218

[15] Kotecha D Asselbergs FW Achenbach S Anker SD Atar D Baigent C . CODE-EHR best-practice framework for the use of structured electronic healthcare records in clinical research. BMJ. (2022) 378:e069048. doi: 10.1136/bmj-2021-069048, 36562446 PMC9403753

[16] Centers for Disease Control and Prevention. Laboratory testing for *Mycoplasma pneumoniae*. (2026). Available online at: https://www.cdc.gov/mycoplasma/php/laboratories/index.html (accessed June 24, 2026).

[17] Centers for Disease Control and Prevention. *Mycoplasma pneumoniae* infection surveillance and trends. (2026). Available online at: https://www.cdc.gov/mycoplasma/php/surveillance/index.html (accessed June 24, 2026).

[18] Yang S Lu S Guo Y Luan W Liu J Wang L. A comparative study of general and severe *Mycoplasma pneumoniae* pneumonia in children. BMC Infect Dis. (2024) 24:449. doi: 10.1186/s12879-024-09340-x, 38671341 PMC11046970

[19] Zhu Y Luo Y Li L Jiang X Du Y Wang J . Immune response plays a role in *Mycoplasma pneumoniae* pneumonia. Front Immunol. (2023) 14:1189647. doi: 10.3389/fimmu.2023.1189647, 37304280 PMC10250694

[20] Xu X Li H Sheng Y Wu L Wang D Liu L . Nomogram for prediction of bronchial mucus plugs in children with *Mycoplasma pneumoniae* pneumonia. Sci Rep. (2020) 10:4579. doi: 10.1038/s41598-020-61348-w, 32165709 PMC7067858

[21] Chunlian X Lili Z Libin Z Yichun X. Macrolide-resistant *Mycoplasma pneumoniae* is an independent risk factor for bronchial mucus plug formation in children. Eur J Pediatr. (2025) 184:264. doi: 10.1007/s00431-025-06095-8, 40123017 PMC11930860

[22] Har Shefi T Oster Y Gordon O Durst R Michael-Gayego A Hoss S . Cardiac manifestations of *Mycoplasma pneumoniae* infections: a cohort study 2007-2024. Clin Infect Dis. (2025) 81:e269–76. doi: 10.1093/cid/ciaf453, 40887973 PMC12728289

[23] Spuesens EBM Fraaij PLA Visser EG Hoogenboezem T Hop WCJ van Adrichem LNA . Carriage of *Mycoplasma pneumoniae* in the upper respiratory tract of symptomatic and asymptomatic children: an observational study. PLoS Med. (2013) 10:e1001444. doi: 10.1371/journal.pmed.1001444, 23690754 PMC3653782

[24] Yu A Ran L Sun X Feng T. Significance of respiratory virus coinfection in children with *Mycoplasma pneumoniae* pneumonia. BMC Pulm Med. (2024) 24:585. doi: 10.1186/s12890-024-03380-4, 39587500 PMC11590504

[25] Li F Zhang Y Shi P Cao L Su L Fu P . Mycoplasma pneumoniae and adenovirus coinfection cause pediatric severe community-acquired pneumonia. Microbiol Spectrum. (2022) 10:e0002622. doi: 10.1128/spectrum.00026-22, 35311565 PMC9045297

[26] Wu Y Zhu Q Liu J Chuan H He L Wang M . Influenza a virus and *Mycoplasma pneumoniae* coinfection mediates immune dysregulation and exacerbates disease severity. Int J Mol Sci. (2025) 26:10487. doi: 10.3390/ijms262110487, 41226526 PMC12607711

[27] Scotta MC Chakr VC de Moura A Becker RG Pinto LA Jones MH . Respiratory viral coinfection and disease severity in children: a systematic review and meta-analysis. J Clin Virol. (2016) 80:45–56. doi: 10.1016/j.jcv.2016.04.019, 27155055 PMC7185664

