# Supplementary material for: Age-related heterogeneity in diagnosis-coded pediatric Mycoplasma pneumoniae-associated hospitalizations and PICU-associated care: a single-center retrospective study
Source: Front Public Health. 2026 Sep 23;14:1875440. doi: 10.3389/fpubh.2026.1875440 (PMC13642579; doi:10.3389/fpubh.2026.1875440)
Supplement: Supplementary file 1 [file Supplementary_file_1.DOCX]

**Supplementary Table S1. English and source-language grouping rules for selected diagnosis-coded variables.**

| **Grouped diagnosis-coded variable** | **English interpretation of matching terms or rule** | **Original source-language diagnosis strings or rule (Chinese)** |
| --- | --- | --- |
| PICU-associated hospitalization | PICU care recorded at any time during hospitalization according to structured discharge fields | 结构化PICU字段或入院/转科/出院科室显示住院期间接受PICU照护 |
| Respiratory failure / hypoxemia | respiratory failure; hypoxemia | 呼吸衰竭; 低氧血症 |
| ARDS | acute respiratory distress syndrome | ARDS; 急性呼吸窘迫综合征 |
| Severe pneumonia | severe pneumonia | 重症肺炎 |
| Coded sepsis / septicemia | sepsis; septicemia | 脓毒血症; 败血症 |
| Cardiac abnormality / coded myocardial involvement | cardiac abnormality; myocardial injury; myocarditis; abnormal myocardial enzyme profile; ECG abnormality | 心肌损害; 心肌炎; 心肌酶谱异常; 病毒性心肌炎; 心电图异常 |
| Neurologic/CNS-related diagnoses | encephalitis; central nervous system infection; convulsion; loss of consciousness; cerebral dysfunction; febrile seizure; encephalopathy; epilepsy | 脑炎; 中枢神经系统感染; 惊厥; 意识丧失; 大脑功能障碍; 发热性惊厥; 脑病; 癫痫 |
| Hepatic involvement | abnormal liver function; hepatic injury; hepatitis | 肝功能异常; 肝损害; 肝炎 |
| Rash / immune-related diagnoses | rash; urticaria; allergic reaction; purpura; Kawasaki disease; immune-related term; allergic purpura | 皮疹; 荨麻疹; 过敏; 紫癜; 川崎; 免疫; 过敏性紫癜 |
| Gastrointestinal manifestations | diarrhea; vomiting; gastrointestinal diagnosis; enteritis; abdominal pain | 腹泻; 呕吐; 胃肠; 肠炎; 腹痛 |
| Atelectasis | atelectasis | 肺不张 |
| Consolidation | pulmonary consolidation | 肺实变 |
| Pleural effusion | pleural effusion | 胸腔积液 |
| Plastic bronchitis | plastic bronchitis | 塑型性支气管炎 |
| Pneumonia | pneumonia; bronchopneumonia; severe pneumonia; wheezy bronchopneumonia; M. pneumoniae pneumonia; bacterial pneumonia; viral pneumonia; Haemophilus influenzae pneumonia; parainfluenza virus pneumonia; adenovirus pneumonia; respiratory syncytial virus pneumonia; staphylococcal pneumonia; pneumococcal pneumonia; Gram-negative bacterial pneumonia | 肺炎; 支气管肺炎; 重症肺炎; 喘息性支气管肺炎; 肺炎支原体性肺炎; 细菌性肺炎; 病毒性肺炎; 流感嗜血杆菌性肺炎; 副流感病毒肺炎; 腺病毒肺炎; 呼吸道合胞体病毒肺炎; 葡萄球菌性肺炎; 肺炎链球菌性肺炎; 革兰阴性细菌性肺炎 |
| Bronchitis | acute bronchitis; bronchitis; acute wheezy bronchitis; acute suppurative bronchitis; rhinovirus acute bronchitis; parainfluenza virus acute bronchitis; respiratory syncytial virus acute bronchitis; M. pneumoniae acute bronchitis | 急性支气管炎; 支气管炎; 急性喘息性支气管炎; 急性化脓性支气管炎; 鼻病毒急性支气管炎; 副流感病毒急性支气管炎; 呼吸道合胞体病毒急性支气管炎; 肺炎支原体急性支气管炎 |
| Bronchiolitis | bronchiolitis; small-airway bronchiolitis | 毛细支气管炎; 细支气管炎 |
| Leukopenia | leukopenia | 白细胞减少 |
| Neutropenia | neutropenia | 中性粒细胞减少 |
| Thrombocytopenia | thrombocytopenia | 血小板减少 |
| Shock | shock | 休克 |
| Pneumothorax / mediastinal emphysema | pneumothorax; mediastinal emphysema | 气胸; 纵隔气肿 |

Note. Original Chinese diagnosis strings are retained only to document the source-language coding algorithm and improve reproducibility. Each hospitalization record was counted once for a grouped variable if one or more corresponding discharge diagnosis labels were present; repeated labels within the same hospitalization record were not counted more than once.

**Supplementary Table S2. Monthly diagnosis-coded M. pneumoniae-associated hospitalization counts and PICU-associated burden.**

| **Month** | **Total M. pneumoniae-associated hospitalization records (n)** | **PICU-associated records (n)** | **PICU-associated rate (%)** |
| --- | --- | --- | --- |
| 2020-06 | 20 | 0 | 0.00 |
| 2020-07 | 122 | 0 | 0.00 |
| 2020-08 | 115 | 1 | 0.87 |
| 2020-09 | 145 | 1 | 0.69 |
| 2020-10 | 240 | 4 | 1.67 |
| 2020-11 | 239 | 2 | 0.84 |
| 2020-12 | 245 | 5 | 2.04 |
| 2021-01 | 208 | 4 | 1.92 |
| 2021-02 | 84 | 0 | 0.00 |
| 2021-03 | 140 | 2 | 1.43 |
| 2021-04 | 270 | 3 | 1.11 |
| 2021-05 | 296 | 1 | 0.34 |
| 2021-06 | 282 | 7 | 2.48 |
| 2021-07 | 310 | 0 | 0.00 |
| 2021-08 | 179 | 3 | 1.68 |
| 2021-09 | 173 | 0 | 0.00 |
| 2021-10 | 292 | 3 | 1.03 |
| 2021-11 | 368 | 3 | 0.82 |
| 2021-12 | 383 | 2 | 0.52 |
| 2022-01 | 287 | 2 | 0.70 |
| 2022-02 | 146 | 1 | 0.68 |
| 2022-03 | 207 | 1 | 0.48 |
| 2022-04 | 276 | 7 | 2.54 |
| 2022-05 | 255 | 1 | 0.39 |
| 2022-06 | 423 | 6 | 1.42 |
| 2022-07 | 536 | 9 | 1.68 |
| 2022-08 | 361 | 8 | 2.22 |
| 2022-09 | 214 | 7 | 3.27 |
| 2022-10 | 264 | 8 | 3.03 |
| 2022-11 | 102 | 5 | 4.90 |
| 2022-12 | 137 | 5 | 3.65 |
| 2023-01 | 99 | 2 | 2.02 |
| 2023-02 | 139 | 1 | 0.72 |
| 2023-03 | 418 | 0 | 0.00 |
| 2023-04 | 441 | 7 | 1.59 |
| 2023-05 | 531 | 18 | 3.39 |
| 2023-06 | 751 | 13 | 1.73 |
| 2023-07 | 1102 | 28 | 2.54 |
| 2023-08 | 1299 | 16 | 1.23 |
| 2023-09 | 1277 | 42 | 3.29 |
| 2023-10 | 2001 | 99 | 4.95 |
| 2023-11 | 2282 | 71 | 3.11 |
| 2023-12 | 2006 | 72 | 3.59 |
| 2024-01 | 1544 | 58 | 3.76 |
| 2024-02 | 818 | 36 | 4.40 |
| 2024-03 | 855 | 30 | 3.51 |
| 2024-04 | 965 | 31 | 3.21 |
| 2024-05 | 1132 | 52 | 4.59 |
| 2024-06 | 1032 | 36 | 3.49 |
| 2024-07 | 879 | 31 | 3.53 |
| 2024-08 | 544 | 20 | 3.68 |
| 2024-09 | 435 | 13 | 2.99 |
| 2024-10 | 494 | 15 | 3.04 |
| 2024-11 | 473 | 16 | 3.38 |
| 2024-12 | 498 | 7 | 1.41 |
| 2025-01 | 400 | 17 | 4.25 |
| 2025-02 | 192 | 5 | 2.60 |
| 2025-03 | 289 | 6 | 2.08 |
| 2025-04 | 300 | 3 | 1.00 |
| 2025-05 | 259 | 5 | 1.93 |
| 2025-06 | 251 | 7 | 2.79 |
| 2025-07 | 248 | 1 | 0.40 |
| 2025-08 | 167 | 2 | 1.20 |
| 2025-09 | 227 | 1 | 0.44 |
| 2025-10 | 257 | 4 | 1.56 |
| 2025-11 | 289 | 2 | 0.69 |
| 2025-12 | 319 | 2 | 0.63 |
| 2026-01 | 240 | 0 | 0.00 |
| 2026-02 | 156 | 1 | 0.64 |
| 2026-03 | 119 | 1 | 0.84 |

Note. The PICU-associated rate was calculated as the monthly number of PICU-associated hospitalization records divided by total monthly M. pneumoniae-associated hospitalization records.

**Supplementary Table S3. Specific pathogen-related coded diagnosis groups in the full cohort.**

| **Specific pathogen-related coded diagnosis group** | **Standard English diagnosis-string interpretation** | **Original source-language diagnosis strings (Chinese)** | **Cases counted once per hospitalization record** | **Percent of cohort** |
| --- | --- | --- | --- | --- |
| Respiratory syncytial virus | respiratory syncytial virus infection; respiratory syncytial virus pneumonia; respiratory syncytial virus acute bronchitis | 呼吸道合胞病毒感染; 呼吸道合体病毒感染; 呼吸道合胞体病毒感染; 呼吸道合胞病毒肺炎; 呼吸道合胞体病毒肺炎; 呼吸道合胞病毒急性支气管炎; 呼吸道合胞体病毒急性支气管炎 | 2096 | 6.34% |
| Rhinovirus | rhinovirus infection; rhinovirus acute bronchitis | 鼻病毒感染; 鼻病毒急性支气管炎 | 2031 | 6.15% |
| Haemophilus influenzae | Haemophilus influenzae infection; Haemophilus influenzae pneumonia; Haemophilus influenzae as a cause of diseases classified elsewhere | 流感嗜血杆菌感染; 流感嗜血杆菌性肺炎; 流感嗜血杆菌作为分类于其他章疾病的原因 | 1351 | 4.09% |
| Adenovirus | adenovirus infection; adenovirus pneumonia; adenovirus encephalitis | 腺病毒感染; 腺病毒肺炎; 腺病毒性脑炎 | 1348 | 4.08% |
| Influenza virus | influenza; influenza due to identified virus; influenza A(H1N1); influenza with pneumonia; influenza with other respiratory manifestations; influenza with other manifestations; seasonal influenza virus identified; other influenza virus identified; influenza-like acute upper respiratory infection due to identified virus | 流行性感冒; 已知病毒的流行性感冒; 甲型H1N1流行性感冒; 流行性感冒伴有肺炎; 流行性感冒伴有其他呼吸道表现; 流行性感冒伴有其他表现; 甲型H1N1流行性感冒性肺炎; 季节性流感病毒被标明; 其他流感病毒被标明; 已知病毒的流感性急性上呼吸道感染 | 956 | 2.89% |
| Parainfluenza virus | parainfluenza virus infection; parainfluenza virus acute bronchitis; parainfluenza virus pneumonia | 副流感病毒感染; 副流感病毒急性支气管炎; 副流感病毒肺炎 | 803 | 2.43% |
| Streptococcus/Pneumococcus | streptococcal infection; streptococcal infection syndrome; acute post-streptococcal glomerulonephritis; streptococcal pneumonia; pneumococcal infection; Streptococcus pneumoniae as a cause of diseases classified elsewhere; Streptococcus pneumoniae pneumonia; Streptococcus pneumoniae septicemia | 链球菌感染; 链球菌感染综合征; 急性链球菌感染后肾小球肾炎; 链球菌性肺炎; 肺炎球菌感染; 肺炎链球菌作为分类于其他章疾病的原因; 肺炎链球菌性肺炎; 肺炎链球菌性败血症 | 708 | 2.14% |
| Epstein-Barr virus | Epstein-Barr virus infection; Epstein-Barr virus infectious mononucleosis | EB病毒感染; EB病毒性单核细胞增多症 | 614 | 1.86% |
| Chlamydia spp. | Chlamydia infection; Chlamydia pneumonia | 衣原体感染; 衣原体肺炎 | 444 | 1.34% |
| Pertussis | pertussis; pertussis encephalopathy; pertussis pneumonia; Bordetella pertussis pertussis | 百日咳; 百日咳脑病; 百日咳肺炎; 百日咳博德特杆菌性百日咳 | 268 | 0.81% |
| SARS-CoV-2/coronavirus | SARS-CoV-2 infection; coronavirus infection; COVID-19 | 新型冠状病毒感染; 冠状病毒感染; COVID-19; COVID 19 | 141 | 0.43% |
| Cytomegalovirus | cytomegalovirus disease; cytomegalic inclusion disease; cytomegalovirus infection; CMV infection | 巨细胞病毒病; 巨细胞包涵体病; 巨细胞病毒感染; CMV感染 | 125 | 0.38% |
| Staphylococcus spp. | staphylococcal infection; Staphylococcus aureus infection; staphylococcal pneumonia; MRSA infection; methicillin-susceptible Staphylococcus aureus infection; Staphylococcus aureus as a cause of diseases classified elsewhere; Staphylococcus aureus enteritis; Staphylococcus epidermidis septicemia | 葡萄球菌感染; 金黄色葡萄球菌感染; 葡萄球菌性肺炎; 耐甲氧西林金黄色葡萄球菌感染; 耐甲氧西林金黄色葡萄球菌(MRSA); 甲氧西林敏感金黄色葡萄球菌感染; 金黄色葡萄球菌作为分类于其他章疾病的原因; 金黄色葡萄球菌肠炎; 表皮葡萄球菌败血症 | 108 | 0.33% |
| Norovirus | norovirus acute gastroenteropathy; norovirus infection | 诺如病毒性急性胃肠病; 诺如病毒感染 | 96 | 0.29% |
| Fungal infection | fungal infection; pulmonary fungal infection; cutaneous fungal infection; fungal pneumonia | 真菌感染; 肺真菌感染; 皮肤真菌感染; 真菌性肺炎 | 96 | 0.29% |
| Herpesvirus / HSV | herpesvirus infection; herpes simplex virus infection; herpetic stomatitis; viral meningoencephalitis; viral meningitis | 疱疹病毒感染; 单纯疱疹病毒感染; 疱疹性口炎病毒病; 病毒性脑膜脑炎; 病毒性脑膜炎 | 80 | 0.24% |
| Rotavirus | rotavirus enteritis; rotavirus infection | 轮状病毒性肠炎; 轮状病毒感染 | 57 | 0.17% |
| Coxsackievirus | coxsackievirus infection | 柯萨奇病毒感染 | 53 | 0.16% |
| Candida spp. | oral candidiasis; candidal stomatitis; respiratory Candida infection; vulvovaginal candidiasis; candidal pneumonia; candidiasis; Candida tropicalis infection | 口腔念珠菌感染; 念珠菌性口炎; 呼吸道念珠菌感染; 念珠菌性阴道炎; 念珠菌性肺炎; 念珠菌病; 热带白色念珠菌感染 | 40 | 0.12% |
| Salmonella spp. | Salmonella enteritis; Salmonella infection; group B Salmonella enteritis; Salmonella septicemia; Salmonella typhimurium enteritis; Salmonella typhimurium infection; Salmonella sepsis | 沙门菌肠炎; 沙门菌感染; B群沙门菌肠炎; 沙门菌败血症; 鼠伤寒沙门菌肠炎; 鼠伤寒沙门菌感染; 沙门菌脓毒血症 | 38 | 0.11% |
| Pseudomonas aeruginosa | Pseudomonas aeruginosa infection | 铜绿假单胞菌感染 | 23 | 0.07% |
| Klebsiella spp. | Klebsiella pneumoniae infection; Klebsiella infection | 肺炎克雷伯杆菌感染; 肺炎克雷伯菌感染; 克雷伯杆菌感染; 克雷伯菌感染 | 22 | 0.07% |
| Enterovirus | enterovirus infection; viral intestinal infection | 肠病毒感染; 肠道病毒感染; 病毒性肠道感染 | 18 | 0.05% |
| Acinetobacter baumannii | Acinetobacter baumannii infection | 鲍曼不动杆菌感染 | 14 | 0.04% |
| Helicobacter pylori | Helicobacter pylori infection | 幽门螺旋杆菌感染 | 8 | 0.02% |
| Escherichia coli | Escherichia coli infection | 大肠杆菌感染 | 5 | 0.02% |
| Aspergillus spp. | pulmonary aspergillosis; aspergillus pneumonia | 肺曲霉菌病; 曲霉菌性肺炎 | 4 | 0.01% |
| Pneumocystis jirovecii | Pneumocystis jirovecii pneumonia | 卡氏肺孢子菌肺炎 | 3 | 0.01% |
| Enterococcus | Enterococcus faecium infection; enterococcal infection | 屎肠球菌感染; 肠球菌感染 | 3 | 0.01% |
| Parvovirus B19 | parvovirus B19 infection; parvovirus infection | 细小病毒B19感染; 细小病毒感染 | 3 | 0.01% |
| Stenotrophomonas maltophilia | Stenotrophomonas maltophilia bloodstream infection | 嗜麦芽窄食单胞菌感染性菌血症 | 2 | 0.01% |
| Non-tuberculous mycobacteria | pulmonary non-tuberculous mycobacterial disease | 肺非结核分枝杆菌病 | 2 | 0.01% |
| Proteus spp. | Proteus infection | 变形杆菌感染 | 1 | 0.00% |

Note. Each pathogen group was counted once per hospitalization record if one or more corresponding pathogen-specific diagnosis labels were present. Non-specific labels such as bacterial infection, viral infection, infectious fever, respiratory tract infection, and pulmonary infection were not included in the any-pathogen summary variable. Original Chinese diagnosis strings are retained only to document the source-language coding algorithm and improve reproducibility; clinical interpretation should be based on the standard English interpretation column.

**Supplementary Table S4. Most frequent exact non-Mycoplasma co-diagnosis terms.**

| **Rank** | **English interpretation** | **Original source-language exact diagnosis term (Chinese)** | **Cases** | **Percent of cohort** |
| --- | --- | --- | --- | --- |
| 1 | Pneumonia | 肺炎 | 18687 | 56.55% |
| 2 | Bacterial infection | 细菌性感染 | 5585 | 16.90% |
| 3 | Viral infection | 病毒性感染 | 5528 | 16.73% |
| 4 | Atelectasis | 肺不张 | 4756 | 14.39% |
| 5 | Sinusitis | 鼻窦炎 | 4225 | 12.78% |
| 6 | Respiratory tract infection | 呼吸道感染 | 3794 | 11.48% |
| 7 | Ventilatory dysfunction | 通气功能障碍 | 3674 | 11.12% |
| 8 | Sepsis / septicemia | 脓毒血症 | 2774 | 8.39% |
| 9 | Pulmonary infection | 肺部感染 | 2679 | 8.11% |
| 10 | Electrocardiographic abnormality | 心电图异常 | 2678 | 8.10% |
| 11 | Coded myocardial injury | 心肌损害 | 2637 | 7.98% |
| 12 | Pulmonary consolidation | 肺实变 | 2449 | 7.41% |
| 13 | Abnormal pulmonary function test result | 肺功能检查的异常结果 | 2271 | 6.87% |
| 14 | Acute bronchitis | 急性支气管炎 | 2095 | 6.34% |
| 15 | Respiratory syncytial virus infection | 呼吸道合胞病毒感染 | 2067 | 6.25% |
| 16 | Rhinovirus infection | 鼻病毒感染 | 2029 | 6.14% |
| 17 | Elevated lactate dehydrogenase | 乳酸脱氢酶升高 | 2021 | 6.12% |
| 18 | Infectious fever | 感染性发热 | 1654 | 5.00% |
| 19 | Adenoid hypertrophy | 腺样体肥大 | 1544 | 4.67% |
| 20 | Severe pneumonia | 重症肺炎 | 1501 | 4.54% |

Note. Terms are ranked by hospitalization-record count, not by raw term frequency alone. The original Chinese term is retained because it is the source-language discharge diagnosis string; the English interpretation is provided for readability.

**Supplementary Table S5. Parsimonious exploratory logistic regression model for PICU-associated hospitalization.**

| **Variable** | **Adjusted OR (95% CI)** | **P value** |
| --- | --- | --- |
| Age: 1-<12 mo* vs 72-<144 mo | 2.45 (1.98-3.03) | <0.001 |
| Age: 12-<36 mo vs 72-<144 mo | 1.34 (1.11-1.61) | 0.002 |
| Age: 36-<72 mo vs 72-<144 mo | 1.03 (0.86-1.23) | 0.775 |
| Age: 144-<216 mo vs 72-<144 mo | 1.13 (0.67-1.92) | 0.644 |
| Male sex | 1.14 (1.00-1.31) | 0.058 |
| Rhinovirus | 0.88 (0.65-1.18) | 0.397 |
| Haemophilus influenzae | 1.03 (0.74-1.45) | 0.842 |
| Adenovirus | 1.35 (0.99-1.85) | 0.060 |
| Respiratory syncytial virus | 1.41 (1.12-1.78) | 0.004 |
| Influenza virus | 1.99 (1.46-2.71) | <0.001 |

Note. This model included age group, sex, and selected pathogen-related coded co-diagnosis variables. Age is expressed in months; mo = months. The 1-<12 mo group began at age 29 days because records from children aged <29 days were excluded. The model should not be interpreted as a causal prediction model.

**Supplementary Table S6. Major chronic or congenital comorbidity categories in the final analytic cohort.**

| **Diagnosis-coded comorbidity category** | **Overall** | **PICU-associated hospitalization** | **Non-PICU hospitalization** |
| --- | --- | --- | --- |
| Any major chronic/congenital comorbidity | 1155 (3.5%) | 172 (19.7%) | 983 (3.1%) |
| Chromosomal / syndromic disorder | 407 (1.2%) | 41 (4.7%) | 366 (1.1%) |
| Congenital heart disease | 247 (0.7%) | 72 (8.3%) | 175 (0.5%) |
| Congenital airway / lung malformation | 330 (1.0%) | 36 (4.1%) | 294 (0.9%) |
| Epilepsy / seizure disorder | 103 (0.3%) | 32 (3.7%) | 71 (0.2%) |
| Metabolic disease | 88 (0.3%) | 17 (1.9%) | 71 (0.2%) |
| Malignancy / hematologic disorder | 33 (0.1%) | 11 (1.3%) | 22 (0.1%) |
| Cerebral palsy | 21 (0.1%) | 2 (0.2%) | 19 (0.1%) |
| Immunodeficiency | 15 (0.0%) | 3 (0.3%) | 12 (0.0%) |
| Bronchopulmonary dysplasia | 3 (0.0%) | 2 (0.2%) | 1 (0.0%) |
| Congenital gastrointestinal malformation | 5 (0.0%) | 4 (0.5%) | 1 (0.0%) |

Note. Comorbidity categories were derived from diagnosis-coded discharge labels and were not mutually exclusive.

**Supplementary Table S7A. Extended exploratory logistic regression model for PICU-associated hospitalization.**

| **Variable** | **Adjusted OR (95% CI)** | **P value** |
| --- | --- | --- |
| Age: 1-<12 mo* vs 72-<144 mo | 2.59 (2.07-3.24) | <0.001 |
| Age: 12-<36 mo vs 72-<144 mo | 1.70 (1.40-2.07) | <0.001 |
| Age: 36-<72 mo vs 72-<144 mo | 1.15 (0.95-1.38) | 0.146 |
| Age: 144-<216 mo vs 72-<144 mo | 1.10 (0.64-1.87) | 0.732 |
| Admission year: 2020 vs 2024 | 0.16 (0.09-0.29) | <0.001 |
| Admission year: 2021 vs 2024 | 0.17 (0.12-0.26) | <0.001 |
| Admission year: 2022 vs 2024 | 0.42 (0.32-0.56) | <0.001 |
| Admission year: 2023 vs 2024 | 0.80 (0.68-0.94) | 0.006 |
| Admission year: 2025 vs 2024 | 0.36 (0.26-0.48) | <0.001 |
| Admission year: 2026 vs 2024 | 0.07 (0.02-0.28) | <0.001 |
| Season: Spring vs Autumn | 0.66 (0.54-0.81) | <0.001 |
| Season: Summer vs Autumn | 0.65 (0.54-0.79) | <0.001 |
| Season: Winter vs Autumn | 0.78 (0.64-0.95) | 0.012 |
| Male sex | 1.11 (0.97-1.28) | 0.127 |
| Rhinovirus | 0.79 (0.58-1.07) | 0.121 |
| Haemophilus influenzae | 0.93 (0.65-1.31) | 0.662 |
| Adenovirus | 1.17 (0.85-1.62) | 0.344 |
| Respiratory syncytial virus | 1.83 (1.43-2.35) | <0.001 |
| Influenza virus | 2.07 (1.50-2.86) | <0.001 |
| Any major chronic/congenital comorbidity | 8.07 (6.69-9.74) | <0.001 |

Note. This extended exploratory model included age group, sex, selected pathogen-related coded co-diagnosis variables, any major chronic/congenital comorbidity, admission year, and season. Season was assigned according to admission month: spring = March-May, summer = June-August, autumn = September-November, and winter = December-February. The reference categories were 72-<144 mo for age group, 2024 for admission year, and autumn for season. This model should not be interpreted as a causal prediction model. OR, odds ratio; CI, confidence interval; PICU, pediatric intensive care unit; mo, months.

**Supplementary Table S7B. Model diagnostic summary for the extended exploratory model.**

| **Model diagnostic** | **Value** |
| --- | --- |
| C-statistic / AUC | 0.698 |
| Brier score | 0.025 |
| Outcome events included in model | 872 |
| Total hospitalization records included in model | 33047 |
| Reference age group | 72-<144 mo |
| Reference admission year | 2024 |
| Reference season | Autumn |

Note. Diagnostics refer to the extended exploratory logistic regression model in Supplementary Table S7A.

**Supplementary Table S8. Microbiological evidence distribution in the stratified chart-validation subset.**

| **Microbiological evidence category** | **Overall validation subset (n=762)** | **PICU validation subset (n=247)** | **Non-PICU validation subset (n=515)** |
| --- | --- | --- | --- |
| Any molecular evidence (PCR/NAAT, tNGS, mNGS, or nanopore) | 737 (96.7%) | 242 (98.0%) | 495 (96.1%) |
| PCR/NAAT evidence | 565 (74.1%) | 165 (66.8%) | 400 (77.7%) |
| tNGS evidence | 136 (17.8%) | 41 (16.6%) | 95 (18.4%) |
| mNGS evidence | 36 (4.7%) | 36 (14.6%) | 0 (0.0%) |
| Nanopore evidence | 0 (0.0%) | 0 (0.0%) | 0 (0.0%) |
| Supportive single-serology evidence | 715 (93.8%) | 212 (85.8%) | 503 (97.7%) |
| Paired serology or four-fold rise | 25 (3.3%) | 5 (2.0%) | 20 (3.9%) |
| Molecular plus serological evidence | 702 (92.1%) | 209 (84.6%) | 493 (95.7%) |
| Serology-only evidence | 25 (3.3%) | 5 (2.0%) | 20 (3.9%) |
| Missing or unclear microbiological evidence | 0 (0.0%) | 0 (0.0%) | 0 (0.0%) |

Note. Categories were derived from the 762-record stratified chart-validation subset. Single-serology evidence was treated as supportive rather than definitive evidence.

**Supplementary Table S9A. Chart-validation summary for selected coded variables.**

| **Validation item** | **Overall validation subset (n=762)** | **PICU validation subset (n=247)** | **Non-PICU validation subset (n=515)** |
| --- | --- | --- | --- |
| Coded pulmonary/extrapulmonary phenotype supported by chart review | 761 (99.9%) | 247 (100.0%) | 514 (99.8%) |
| Major chronic/congenital comorbidity coding concordant with chart review | 722 (94.8%) | 216 (87.4%) | 506 (98.3%) |
| Additional respiratory pathogen documented as detected | 632 (82.9%) | 188 (76.1%) | 444 (86.2%) |

Note. Validation items were summarized in the 762-record stratified chart-validation subset.

**Supplementary Table S9B. PICU-associated care reasons in the PICU validation subset.**

| **PICU-associated care reason category (PICU validation subset only)** | **PICU validation subset (n=247)** |
| --- | --- |
| Respiratory failure and/or hypoxemia-related PICU reason | 198 (80.2%) |
| Severe pneumonia-related PICU reason | 102 (41.3%) |
| Sepsis-related PICU reason | 28 (11.3%) |
| Convulsion/coma/neurologic monitoring-related PICU reason | 26 (10.5%) |
| Shock-related PICU reason | 5 (2.0%) |
| Heart failure-related PICU reason | 2 (0.8%) |
| Other documented PICU-associated reason | 3 (1.2%) |

Note. PICU-associated care reason categories were assessed only among PICU validation subset records (n=247). Categories are not mutually exclusive.
